# Modulation of Multiple Precipitates for High Strength and Ductility in Al-Cu-Mn Alloy

**DOI:** 10.3390/ma14237383

**Published:** 2021-12-02

**Authors:** Linxiang Liu, Zhijun Wang, Qingfeng Wu, Zhongsheng Yang, Kexuan Zhou, Xiaoguang Fan, Junjie Li, Jincheng Wang

**Affiliations:** State Key Laboratory of Solidification Processing, Northwestern Polytechnical University, Xi’an 710072, China; lxliu@mail.nwpu.edu.cn (L.L.); qingfengwu@mail.nwpu.edu.cn (Q.W.); zsyang@mail.nwpu.edu.cn (Z.Y.); kx_zhou@mail.nwpu.edu.cn (K.Z.); fxg3200@nwpu.edu.cn (X.F.); lijunjie@nwpu.edu.cn (J.L.)

**Keywords:** aluminum alloys, pre-deformation, mechanical properties, strengthening mechanisms

## Abstract

The category and morphology of precipitates are essential factors in determining the mechanical behaviors of aluminum alloys. It is a great challenge to synthetically modulate multiple precipitates to simultaneously improve strength and ductility. In the present work, by optimizing the precipitations of the GP zone, θ’-approximant and θ’ phase for an Al-Cu-Mn alloy, a high tensile strength of 585 MPa with large elongation of 12.35% was achieved through pre-deformation and aging. The microstructure evolution pattern was revealed by detailed characterizations of scanning electron microscopy and transmission electron microscopy. It was found that such high tensile strength of the samples was due to a combination of strengthening by the high density of dispersive fine precipitates and dislocations, and the high elongation to failure was primarily attributed to the multimodal precipitates and elimination of precipitation-free zones along the grain boundaries. The strategy proposed here is a promising way of preparing ultra-strong Al-Cu-Mn alloys.

## 1. Introduction

Aluminum–copper alloys have been widely developed and used in the fields of aerospace and transportation due to their low density and high strength [1]. Typically, as an Al-Cu-Mn alloy, the 2219 alloy is extensively exploited and used as a structural material in the aerospace industry, such as transition rings constituting a tube, short shells, and a dome of tanks, because of its excellent mechanical properties over a wide temperature range and good weldability [2]. Similarly, with a proper combination of mechanical properties, ballistic resistance and resistance to stress corrosion cracking, the 2519 alloy and its upgrade version, 2519A, have been widely used in naval structures, such as advanced amphibious assault vehicles (AAAVs) [3]. Similar to the 2219 alloy, the ZL205A alloy is a casting Al-Cu-Mn alloy that has outstanding mechanical properties, and it has been widely applied in aircraft frame components. Its ultimate tensile strength can exceed 500 MPa after T6 treatment. To date, most of the research has focused on the traditional microalloying [4,5,6] or optimization of the solidification process [7,8,9,10,11] to further improve the strength of ZL205A as a casting alloy.

For ultra-strong aluminum alloy, ultra-fine grains (UFGs) produced by severe plastic deformation (SPD) are attracting increasing attention [12]. Numerous methods for SPD processing are now available for wrought aluminum alloy, such as equal-channel angular pressing (ECAP) [13,14,15], high pressure torsion (HPT) [16], cryorolling [17] and accumulative roll bonding (ARB) [18]. Guo et al. [19] systematically investigated the evolution of microstructure of the ZL205A alloy during ECAP at room temperature, illustrating that the hardness of the heat-treated ZL205A alloy increased from 112 HV to 198 HV after two passes of ECAP. However, in the consideration of the diminishing strain hardening capacity in UFG materials, low ductility under ambient conditions would be the challenge to be overcome [20,21,22]. 

In contrast, thermo-mechanical treatment (TMT) such as the T8 series has caught increasing attention in industrial manufacturing. For instance, the mechanical performances of Al-Cu-Mn alloys can be improved by tailoring their precipitation behaviors in TMT. In addition, it has been reported that pre-deformation can optimize aging precipitation behavior and improve the strength of the 2219 alloy [23,24,25]. Li et al. [26] subjected 2219 alloy samples to 8.0% pre-stretch before aging, resulting in a 22% increase in yield strength because of the deformation-ameliorated precipitation distribution. When the pre-deformation degree was increased to 80%, a 130 MPa strength improvement without loss of ductility came to the heavily rolled Al-Cu alloy after subsequent low-temperature aging. This is due to the dislocation hardening and precipitation strengthening by a kind of fine coherent θ’-approximant [27]. The nucleation sites from the generated dislocation in the pre-deformation step can promote precipitation or even change the sequences of multiple precipitates. 

To date, the precipitation behavior in the ZL205A alloy under TMT has lacked attention. The appropriate aging treatment with pre-deformation could be an effective way of designing the bimodal microstructure of precipitation in Al-Cu alloys [28]. In view of the ZL205A alloy being a candidate for a structural material with high strength and ductility, we obtained excellent strength and ductility in the ZL205A alloy by developing a TMT procedure to control precipitation behaviors. Both ductility and strength are superior among the reported Al-Cu-Mn alloys. The strategy was systematically optimized by adjusting the solution treatment, large cold deformation and aging treatment. The microstructure evolution was analyzed using scanning electron microscopy (SEM) and transmission electron microscopy (TEM). 

## 2. Experiments

In this work, a commercial ZL205A alloy ingot is used, whose composition is listed in Table 1. The ingot was firstly solution treated at 538 °C for 15 h and then water-quenched into room temperature (RT) to form the solid solution samples with coarse grains. Then, a conventional T6 treatment was applied for comparison (aging at 160 °C). For TMT, the thickness reductions for RT cold rolling were 70%, 80% and 90%, respectively. Different aging conditions were then applied at 120 °C or 160 °C for 0–24 h to optimize the strength/ductility combination.

Vickers hardness (300 g load with 15 s dwell time) measurements were carried out on the cold rolled, annealed and aged samples to assess the strength. At least five data points were recorded to obtain the average hardness value of each sample. Uniaxial tensile tests were performed at strain rates of 10^−3^ s^−1^ on samples that were cut along the rolling direction and polished into dog-bone shapes with a gauge length of 12.5 mm. All the tests were performed at least three times in order to ensure the repeatability and reproducibility of the results.

X-ray diffraction (XRD) measurements (2θ from 20° to 100°) were carried out on Rigakud/max-2550 with a monochromator. The scanning step was 0.02°, and the scanning rate was 5° per min. Microstructure characterization was performed using TEM (JEOL 2100 F, JEOL, Tokyo, Japan) and SEM (Tescan Mira 3, Tescan, Brno, The Czech Republic) equipped with back scattered electron (BSE) detector. The SEM specimens were prepared by grinding the samples with abrasive papers, and, subsequently, they were subjected to electropolishing with the solution of 30% perchloric acid in ethanol. All of the TEM specimens were prepared by twin-jet polishing with a solution of 30% nitric acid and 70% methanol at −30 °C and 20 V.

## 3. Results and Discussion

### 3.1. Evolution of Mechanical Properties during Different Processing Stages

**Figure 1:**
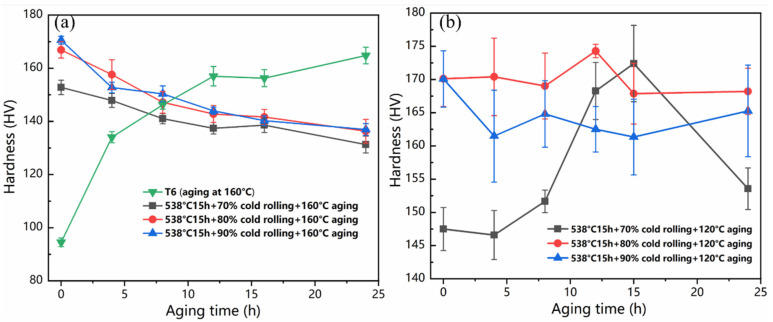
Variation of Vickers hardness during aging for samples subjected to (**a**): T6 and aging at 160 °C after 70–90% cold rolling and (**b**) aging at 120 °C after 70–90% cold rolling.

Figure 1 displays the Vickers hardness variation as a function of time for different treatments on the ZL205A alloy. As shown in Figure 1a, the hardness of the T6processed sample increased to a plateau of ~160 HV after 10 h aging. After large pre-deformation, the hardness of the samples increased sharply, and the largest hardness value of the 90% rolled sample (~172 HV) was much greater than that of the solid solution state. Compared with the conventional T6-processed sample, the hardness decreased rapidly with the extension of aging time at 160 °C. Both 160 °C and 120 °C are common aging temperatures for the ZL205A Al-Cu alloy [27]. Theoretically, aging at 160 °C could accelerate the precipitation kinetics after large pre-deformation, leading to more dispersive fine precipitates and higher hardness at the early stage. However, the hardness of the deformed sample decreases continuously at 160 °C. When heating a largely deformed supersaturated alloy, there will be a competitive phenomenon between recovery/recrystallization and precipitation. In general, at relatively higher temperatures, the recovery/recrystallization starts earlier before there is significant precipitation. Therefore, the reason for the decreasing hardness at the early stage could be interpreted as due to the descent of dislocation density brought by the quicker recovery/recrystallization. A similar phenomenon could also be observed in the results of Ma et al.’s study [27], where a quicker decrease in hardness was found at the same deformation degree. Thus, it could be deduced that the temperature of aging should be relative lower for the sample that underwent large pre-deformation. As shown in Figure 1b, we subsequently aged the sample after large pre-deformation at 120 °C, finding that the samples with different pre-deformation degrees exhibited various evolution tendencies of hardness. When aging at 120 °C, the hardness of the 70% cold-rolled sample increased from 147 HV to the maximum of 172 HV for 15 h, followed by a sharp decrease. Intriguingly, for the 80% cold-rolled sample, the hardness maintained moderate fluctuation with a peak-aged stage (~175 HV) at 12 h. An evident drop occurred before the hardness of the 90% cold-rolled sample fluctuated in a relatively stable range around 160 HV. 

**Figure 2:**
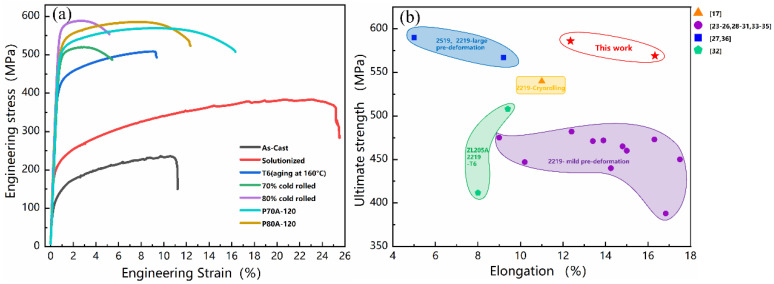
(**a**) Tensile engineering stress–strain curves of samples under different processing states; (**b**) the map of ultimate tensile strength–ductility combinations of existing Al-Cu-Mn alloy research [17,23,24,25,26,27,29,30,31,32,33,34,35,36,37], including our work.

Based on the hardness evolution during the aging process, room-temperature tensile tests were carried out to reveal the evolution of the mechanical properties. Figure 2a plots the typical stress–strain curves of the ZL205A alloy under different processing states. The as-cast sample has a yield strength (YS) of ~110 MPa, an ultimate tensile strength (UTS) of ~240 MPa and an elongation to failure (ETF) close to 11%. After solution treatment at 538 °C for 15 h, both the tensile strength and ductility of the sample were improved. The strength of the peak-aged T6 sample was substantially increased (YS~421 MPa and UTS~508 MPa) by precipitation strengthening, with an ETF close to 9.4%. The strength of 70% and 80% cold-rolled samples significantly increased, while the ductility reduced sharply, especially for the poor uniform elongation, which is typical for cold-worked metals. As the aging temperature for Al-Cu alloy is low, cold rolling could take effect in natural aging to accelerate it. Therefore, besides the high-density dislocations, the hardening effect could partially be attributed to the natural aging in the cold-rolled sample. Generally, and significantly, aging cold-rolled samples can remarkably enhance their tensile ductility while keeping or even modestly increasing strength via precipitation hardening. Therefore, there were excellent mechanical properties of the samples subjected to 70% and 80% cold rolling, which were designated as P70A-120 and P80A-120 for further discussion, respectively. In Figure 2a, it could be found that the ETFs of these two samples were notably increased. The P80A-120 sample yielded higher strength (YS~524 MPa and UTS~585 MPa) than that of the P70A-120 sample (YS~508 MPa and UTS~569 MPa), while its ETF (~12.3%) was slightly lower than that of the P70A-120 sample (~16.3%). Both of these samples exhibited better mechanical properties than those of the T6 sample by overcoming the strength–ductility trade-off. In addition, a comparison of the mechanical properties of the reported Al-Cu-Mn series alloys under various processing methods is illustrated in Figure 2b. The present work presents the superior mechanical properties among the samples subjected to conventional T6 treatment, mild pre-deformation and SPD such as cryorolling.

### 3.2. Evolution of Microstructure in Different Processing Stages

**Figure 3:**
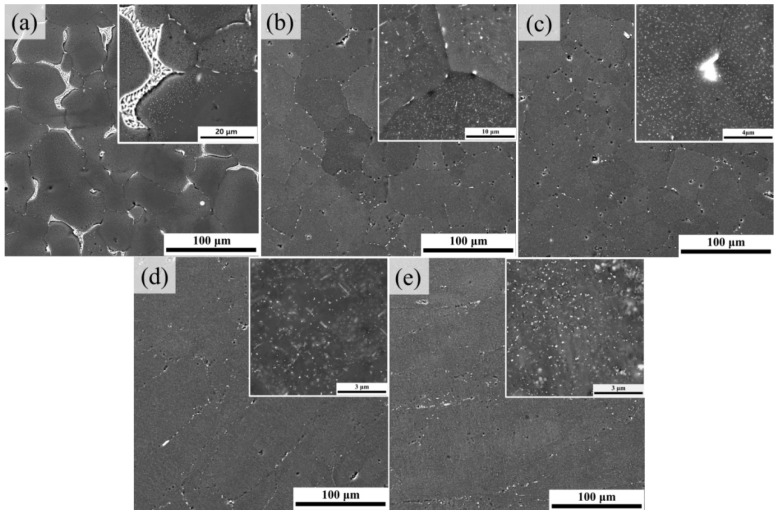
BSE images of the (**a**) as-cast state, (**b**) solutionized state, (**c**) T6 state, (**d**) P70A-120 state and (**e**) P80A-120 state.

**Figure 4:**
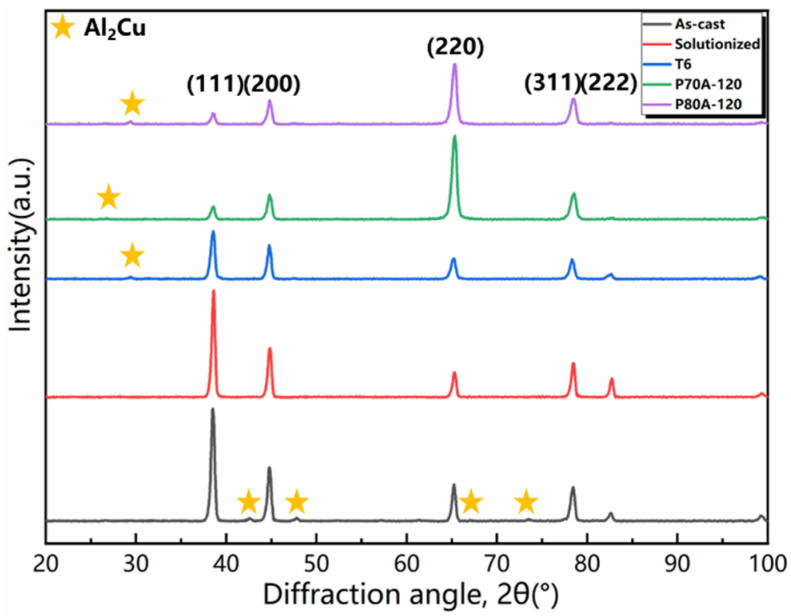
XRD patterns of the as-cast, solutionized, T6, P70A-120 and P80A-120 state.

The microstructure and corresponding XRD patterns of the as-cast, solutionized, T6, P70A-120, and P80A-120 samples are presented in Figure 3 and Figure 4, respectively. Figure 3a demonstrates the microstructure of the as-cast ZL205A alloy containing equiaxed grains (average size ~60 μm). According to the XRD patterns shown in Figure 4, there exist a significant number of eutectic colonies, (Al) + Al_2_Cu, around the grain boundaries, which is consistent with the cast microstructure of the 2219 alloy [38]. The homogenization treatment will cause dissolution of eutectic phases. After solution treatment at 538 °C for 15 h, it can be seen in Figure 3b that most of the eutectics dissolved into the matrix, with a minority remaining at the grain boundaries, in accordance with the disappearance of the peak of the Al_2_Cu phase in the XRD pattern. Furthermore, as shown in the inset of Figure 3b, the rod-like ternary T phase (Al_20_Cu_2_Mn_3_) precipitated inside the grains during homogenization, which effectively promotes the accumulation of dislocations during the large deformation process [39]. In contrast, after conventional T6 treatment, there was a coarse second phase larger than 1 μm located at the triple junction, and many particles were distributed uniformly inside the grains. 

For the P70A-120 (Figure 3d) and P80A-120 (Figure 3e) samples, the grains were stretched along the rolling direction, with the remnant broken second phase distributing along the prolonged grain boundaries. Additionally, the inset of Figure 3d shows that the P70A-120 sample contained numerous fine particles and a certain amount of T phases with a length of approximately 1 μm. Similarly, the inset of Figure 3e shows that massive dispersoids distributed homogeneously all over the grains. According to the XRD patterns plotted in Figure 4, the three peak-aged samples with different pre-deformation degrees had the same diffraction peaks of the Al_2_Cu phase, identifying the second phase particles observed in Figure 3c–e. Moreover, for the T6 sample the strongest peak of the Al matrix in the map appeared at the angle of 38°, while that of the P70A-120 and P80A-120 samples changed to 65°. The strongest peaks of 38° and 65° correspond to the (111) and (220) crystal planes respectively, and the (220) crystal planes equate to the (110) crystal planes. The (111) crystal planes are the glide planes that most likely glide along the < 110 > crystal orientation. The change in crystal planes indicates that the grain was sliding during pre-deformation to some extent [28], and the P70A-120 and P80A-120 samples could have a texture.

**Figure 5:**
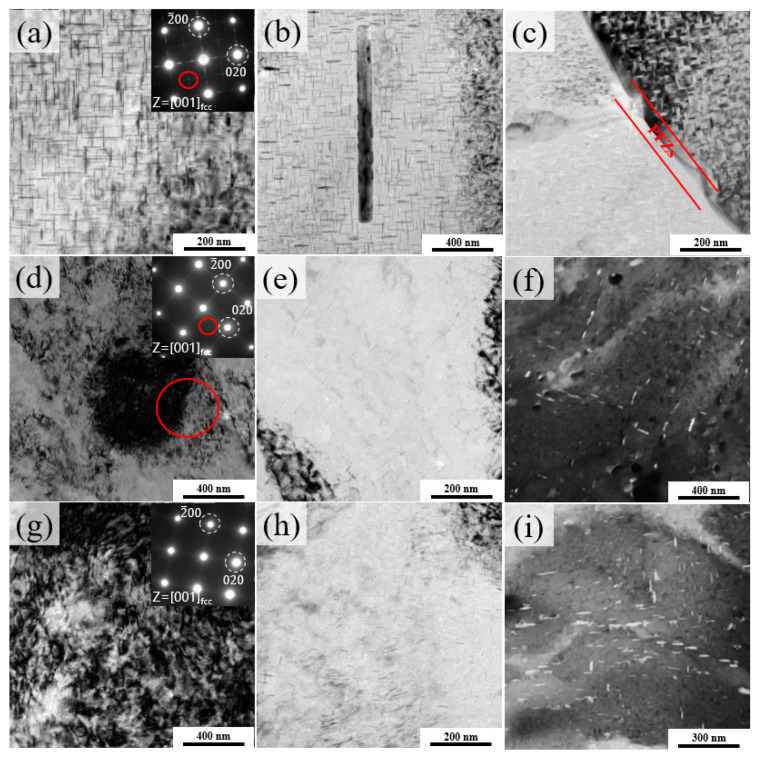
TEM images of the T6 sample: (**a**–**c**) BF image with diffraction pattern; P70A-120 sample: (**d**,**e**) BF image with diffraction pattern and (**f**) STEM image; and P80A-120 sample:(**g**,**h**) BF image with diffraction pattern and (**i**) STEM image.

Figure 5 presents the TEM images of the peak-aged T6, P70A-120 and P80A-120 samples with the electron beam parallel to {001}_Al_. As shown in Figure 5a, there are numerous orthogonal needle-like precipitates widely distributed along the {001}_Al_ planes in the matrix. The corresponding selected area electron diffraction (SAED) pattern in the inset of Figure 5a clearly exhibits additional diffraction patterns at the {110}_Al_ positions, implying that the microstructures of these samples mostly consisted of the θ’ phase. These features are in agreement with previous reports of a θ’ phase formed after T6 treatment of Al-Cu alloys [40,41]. In Figure 5b, the average length and width of the θ’ phases were about 100 nm and 16 nm, respectively. Evidently, the rod-like T-phase had a length and width of approximately 1.3 μm and 0.1 μm, respectively. Additionally, precipitation-free zones (PFZs) about 70 nm wide and coarse Al_2_Cu particles along the grain boundary were observed in the sample subjected to the T6 process at 160 °C, as marked in Figure 5c. The direct reason for the occurrence of PFZs is the lack of solutes inside the grains. In general, after high-temperature solution treatment, alloys contain a high concentration of unstable vacancies, which diffuse to the grain boundaries. Since there is high affinity between vacancies and solute atoms [42], there also exists the solute diffusion accompanied by vacancies, which can explain the lack of solutes inside. 

Figure 5d shows the bright field (BF) image along a zone axis close to {001}_Al_ with a corresponding SAED pattern of the P70A-120 sample. It can be seen that after peak aging the sample still contained a high density of dislocation tangles with a few of the precipitates around them, and the diffraction patterns of the θ’ phase were obscure because of the strain fields of residual dislocations. Moreover, the precipitates were hardly to be observed in the situation with fewer dislocations as marked in Figure 5d. A region with a low density of dislocations was selected as shown in Figure 5e, and there were a few fine but sparse precipitates on the matrix. Similarly, in the view of scanning TEM (STEM) mode (Figure 5f), it could still be observed that those fine precipitates were distributed non-uniformly. In comparison, the density of remnant dislocations was more uniform and denser in the P80A-120 sample (Figure 5g), resulting in the invisibility of SAED patterns of the θ’ phase. The precipitates also became denser at a closer inspection (Figure 5h), indicating that the quantity and uniformity of precipitates increased. In addition, in STEM mode (Figure 5i), it can be observed that the distribution of precipitates was dispersive, and the width of precipitates ranged from 2 nm to 16 nm and the length ranged from 11 nm to 70 nm. In view of the XRD patterns, the observed precipitates with large size in Figure 5f,i could mostly be the θ’ phases.

**Figure 6:**
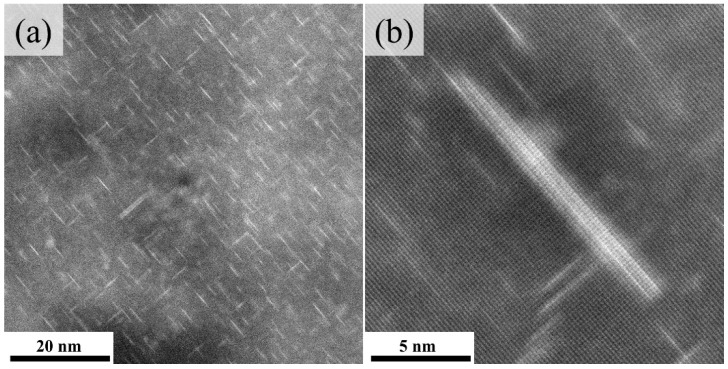
HAADF-STEM images of P80A-120 sample. (**a**) Low-magnification image. (**b**) High-resolution image. The electron beam is parallel to {001}_Al_ direction.

To better characterize the morphology and distribution of the second phase, the precipitates in the P80A-120 sample were characterized in detail by HAADF-STEM. As shown in Figure 6a, the microstructure of the P80A-120 sample consists of a large number of fine precipitates uniformly and densely distributed in the matrix and sparsely dispersed plate-like precipitates. Figure 6b reveals that most of the fine precipitates are GPI zones with a monolayer Cu atomic plane, while the larger precipitate plates can be readily discerned as two Cu-atom layers sandwiching three Al-atom layers lying on {100}_Al_ planes. In addition, the morphology of the larger precipitate plates is consistent with the previous report of a θ’-approximant, which is structurally the same as the θ’ phase except for the absence of a Cu atom in the body center [27,28].

### 3.3. The Effect of Microstructure Evolution on the Mechanical Properties

Based on the results shown above, the majority of remnant dislocations were statistically stored dislocations, and these dislocations could significantly interact with the second phase precipitated heterogeneously [43]. Consequently, the microstructure could be altered considerably by changing the degree of large pre-deformation and aging temperature, leading to the enhancement of the mechanical properties. During the aging process, after cold rolling, microstructure evolution occurs via the following processes: Firstly, the solute atoms precipitate heterogeneously from the supersaturated solid solution to form a metastable phase. Secondly, owing to the annihilation of dislocations, the matrix recovers to soften the alloy. Thirdly, the precipitated particles may exert a significant pinning effect on the dislocations. All three evolutions occur concurrently and compete during the aging process, determining the final microstructure and mechanical properties of the alloy.

It is well proved that deformation-induced dislocations could significantly accelerate and alter precipitation kinetics via short-circuit diffusion [44,45]. Thus, for the P80A-120 sample, multi-stage precipitates could emerge and coarsen after large pre-deformation despite the aging temperature being relatively lower. The ZL205A alloy has a similar chemical composition to that of the 2219 alloy; therefore, after undergoing large pre-deformation, the microstructure of the P80A-120 sample also comprised the θ’-approximant. In addition, the P80A-120 sample has a more θ’ phase than that of the P70A-120 sample because of the larger degree of pre-deformation, as shown in Figure 5f,i.

From the variation in Vickers hardness shown in Figure 1b, it can be deduced that the numerous defects introduced by the 70% degree of pre-deformation could effectively promote the heterogeneous nucleation of precipitates, leading to the continuous enhancement of strength before peak aging. However, with the extension of aging time, the coarsening of the precipitates generally occurred. and the pinning effect declined subsequently, causing a decrease in strength. When the degree of pre-deformation was up to 80%, the dispersive precipitates with higher density could not only compensate for the strength reduction caused by the recovery but also further exert a persistent pinning effect on the movement of dislocations, maintaining the high strength as the rolling state. When the sample underwent 90% degree of pre-deformation, the drop in hardness at the early stage of aging could be ascribed to the fast dislocation annihilation for the higher free energy. 

The tensile yield strength can be expressed using the “composite” Hall–Petch relationship as given below [46]:

(1) $$\sigma_{Y}=\sigma_{0}^{Al}+\sigma_{SS}+\sigma_{GB}+\sigma_{p}+\sigma_{d}$$

where $\sigma_{0}^{Al}$ is the resistance to dislocation glide within the crystallite for pure Al, which was reported to be about 10 MPa [47]; $\sigma_{SS}$ is the solution strengthening caused by dissolved solute atoms; $\sigma_{p}$ is the particle strengthening; and $\sigma_{d}$ and $\sigma_{GB}$ are attributed to dislocation strengthening. Among these factors, $\sigma_{GB}$ is a similar value in all specimens for the reason that the specimens contained the same concentration of elements and were also subjected to the same solution treatment to dissolve the majority of soluble phases. Furthermore, the contribution from GB strengthening to $\sigma_{Y}$ is calculated as below [47]:

(2) $$\sigma_{GB}=k_{y}d_{GB}^{-\frac{1}{2}}$$

where $k_{y}$ is the Hall–Petch slope, which is almost the same in all specimens, and $d_{GB}$ is the distance between boundaries. As shown in Figure 3d,e, after cold rolling, the grains were stretched, and the distance between the extended boundaries was at least twice the solid solution state. Thus, this suggests that the Hall–Petch GB strengthening mechanism term has less contribution to $\sigma_{Y}$ along the rolling direction in the P70A-120 and P80A-120 samples than in the T6 sample.

According to the results of the tensile test (Figure 2a) and the corresponding microstructures (Figure 5 and Figure 6), it can be revealed that achieving the appropriate coexistence of high-density dislocations and dispersive fine precipitates benefits a better strengthening effect from the combination of dislocation hardening ($\sigma_{d}$) and precipitation hardening ($\sigma_{p}$). Among the samples under different processing parameters, the microstructure of the P80A-120 sample has the best matching of the remnant dislocations and a variety of beneficial precipitates, such as θ’, leading to the highest strength among all the samples.

**Figure 7:**
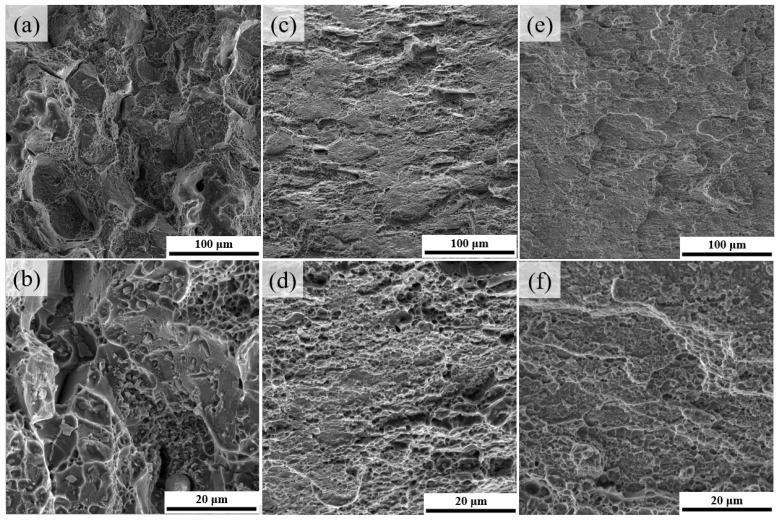
Fracture morphologies of the (**a**,**b**) T6 state, (**c**,**d**) P70A-120 state, and (**e**,**f**) P80A-120 state, corresponding to the samples that underwent uniaxial tensile testing as shown in Figure 4.

The fracture morphologies are shown in Figure 7, revealing the variation in ductility. In the T6 sample, the fracture mode was a mix of intergranular and transgranular, which exhibited a considerable number of intergranular cracks and dimples associated with some coarse dimples. According to the Al-Cu phase diagram, after solution treatment at 538 °C, the Al_2_Cu phase will be in equilibrium with the matrix [48], meaning that the particles observed in fracture surfaces were coarse Al_2_Cu particles that exist in all specimens. However, for the alloy subjected to large pre-deformation, some coarse particles could be broken down and lessened, which effectively reduced the cracking tendency. Furthermore, it can be observed that the number of the particles in the fracture surfaces is high; thus, we cannot attribute these particles to Fe-containing particles since the studied alloy contains a very low amount of Fe. In comparison, Figure 7c–f show that the fracture mode of P70A-120 and P80A-120 were transformed into a mainly transgranular fracture, and the average size of the dimples was smaller. 

The fracture morphologies coincided with the previous analysis of mechanical properties. As shown in Figure 2a, the P70-120 sample and P80-120 presented outstanding ductility in the tensile tests, especially the P70-120 sample, which has nearly twice the ETF than that of the T6 sample. This significant increase in ductility was caused by two optimized microstructures that formed during aging. The first was the decrease in dislocation density because of the recovery, leaving more free paths for dislocation slip and accumulation, which certainly increased the work-hardening rate of the cold-rolled state during tensile testing [39]. The second was the high density of multiple nano-sized precipitates on the matrix without PFZs along the grain boundaries. For the T6 sample, both the weakened PFZs (Figure 5c) and coarse intergranular precipitates (Figure 3c) could easily produce stress concentration near the grain boundaries [49,50]. Significantly, aging after the large degree of pre-deformation could generally prevent the emergence of PFZs along the grain boundaries [51]. Meanwhile, as shown in Figure 6b, the microstructure of the P80A-120 sample has multiple precipitates, such as GP zones, θ’-approximant and θ’, composed of shear-resistant and shearable precipitates to simultaneously increase the yield strength and uniform ductility. For example, the high density of nano-sized θ’ precipitates can provide effective sites for trapping and accumulating dislocations to enhance strength. In addition, the strain-induced dissolution of GP zones and the repartition of solutes to the matrix could contribute to the enhanced strain hardening and, therefore, are responsible for the improvement in plasticity [52]. Finally, the optimized microstructure could sustain the work hardening rate and alleviate the tendency of the intergranular cracking, leading to an improvement in the comprehensive mechanical properties of the ZL205A alloy.

## 4. Conclusions

In summary, we demonstrated a TMT approach for the control of precipitations in a casting Al-Cu-Mn alloy (ZL205A) to simultaneously increase strength and ductility by optimizing the process parameters. The mechanical properties and the microstructures were systematically analyzed. Surprisingly, the mechanical properties obtained here (UTS~ 569 MPa/585 MPa with ETF~16.32%/12.35%) were superior to other wrought Al-Cu-Mn alloys, such as the 2219 alloy. The strengthening and toughening mechanism was investigated via SEM and TEM. In the current study, the optimized TMT for the ZL205A alloy was peak aging at 120 °C after a 70–80% degree of cold rolling in order to induce heterogeneous precipitation and restored dislocations concurrently. The combination of high strength and ductility was ascribed to the formation of multimodal precipitates, including the shearable GP zones/θ’-approximant and shear-resistance θ’. The approach described here could be applicable to other Al alloys whose plasticity of solutionized state allows the conduction of large pre-deformation before aging, even for casting alloys.

## Figures and Tables

**Table 1:** Chemical composition of ZL205A Al-Cu alloy (wt.%).

Cu	Mn	Ti	Zr	Cd	B	V	Si	Fe	Al
4.98	0.39	0.24	0.12	0.21	0.028	0.13	0.021	0.026	Bal

## Data Availability

The data used to support the findings of this study are available from the corresponding author upon request.
